# Timing and Duration of Observation Periods of Foraging Behavior in Natural Grasslands

**DOI:** 10.3389/fvets.2020.519698

**Published:** 2020-11-12

**Authors:** Felipe Jochims, Émerson Mendes Soares, Leandro Bittencourt de Oliveira, Bruno Castro Kuinchtner, Pedro Trindade Casanova, Luciana Marin, Fernando Luiz Ferreira de Quadros

**Affiliations:** ^1^Family Farming Research Center, Santa Catarina Agricultural Research and Rural Extension Company (EPAGRI/CEPAF), Chapecó, Brazil; ^2^Animal Science Department, Federal University of Santa Maria, Santa Maria, Brazil; ^3^Integrated Regional University of Alto Uruguai (URI), Frederico Westphalen, Brazil; ^4^Animal Science Department, Farroupilha Federal Institute, Alegrete, Brazil; ^5^Animal Science Department, Federal University of Santa Maria, Santa Maria, Brazil; ^6^Animal Science Department, Federal University of Santa Maria, Santa Maria, Brazil

**Keywords:** grazing behavior, grazing distribution, foraging activities, monitoring behavior, grazing patterns

## Abstract

The goals of this study were to evaluate the length of time grazing which should be monitored over a 24-h period to predict the grazing behavior of beef heifers within a season and determine the patterns of foraging activity over 24 h. A database was constructed between 2010 and 2012 for beef heifers managed under rotational grazing in a natural grassland. Grazing, rumination, and other activity times were assessed visually during 24 h on 15 occasions. Data were classified according to climatic seasons, generating 12 replicates in summer, 18 in spring, 24 in autumn, and 36 in winter. Treatments were the evaluation of four distinct periods: from sunrise to sunset (DAY-SUN), daylight duration from dawn to nightfall (DAYLIGHT), DAYLIGHT plus 2 h (DAYLIGHT+2), DAYLIGHT to midnight (DAYLIGHT to 0), and the entire 24 h period (CONTROL). Differences for grazing, rumination, and other activities were found in all seasons for the evaluation periods. Sampling sufficiency was reached only with the DAYLIGHT to 0 and CONTROL for all four climatic seasons. The DAYLIGHT to 0 treatment covered 75% of a 24-h period and 95% of the mean foraging time took place during this time interval. Considering grazing distribution during a day, in the warm seasons, the major grazing period during mornings occurred earlier than in the cool seasons, and in cool seasons, the grazing peak was observed during the afternoon. Visual observations from dawn until midnight represented the total grazing time and natural behavior of heifers and could be used to represent grazing activities for the entire day.

## Introduction

Grazing behavior evaluations can be an important issue when establishing management goals because the behavior of animals on pastures provides clues with which to determine if pasture management decisions are suitable (1) and whether animals are expressing their natural behavior, which is a good indication of animal welfare in pasture-based production systems. Furthermore, the behavior of animals in controlled situations, such as grazing trials, can provide insights into the production data collected in those situations.

The southern Brazil Campos grasslands (2) are the major foraging systems for beef cattle reared in this region, and the same is valid for other nearby South American countries, such as Argentina, Uruguay, and part of Paraguay. Thus, developing management tools for these grazing systems can provide productivity gains, as well as ensure the welfare of animals. Grazing systems in these natural grasslands are considered an important marketing advantage for these countries, and monitoring ingestive behavior could attest to the adequate state of animal comfort. Management systems that do not take into account whether animals can attain daily forage intake to meet their nutritional requirements may be inefficient.

Among behavior variables, time spent grazing, and ruminating is the main measured indicative and key variables used as indicators of management efficiency and animal welfare. Other relevant variables of management efficiency could be estimates of dry matter (DM) intake and forage quality that relates to forage on offer and sward structure (3), but these are not animal behavior traits. For example, ruminants commonly have grazing times between 450 and 600 min/day in temperate pastures and rarely forage <360 min/day, with times that may exceed 760 min/day on subtropical and tropical pastures (4, 5). In the southern Brazil Campos grasslands (6), without limitations to inhibit potential intake (e.g., sward height or herbage mass), the time spent grazing commonly ranges between 500 and 650 min/day (7–9), regardless of the grazing method used (10). This variation in grazing time indicates potentially diverse situations and challenges that animals can face, even in situations with abundant forage allowance, attesting to the complex interaction of animals with plants and sward structure. On subtropical natural grasslands, in a wide range of forage allowances, Trindade et al. (11) indicated that both lower and higher levels could limit forage intake, due to limited bite volume and mass at allowances lower than 8 kg of DM/100 kg of liveweight and to limited bite selection above 16 kg of DM/100 kg of liveweight. These bite variations are linked also to grazing time along the day, indicating a “standard range” of 500–600 min/day. However, observed grazing times outside of “standard range” are indicative that something is wrong with animal management, which could even decrease welfare by forcing unusual behavior on animals, such as grazing during hot periods of the day. Notwithstanding, all these protocols of forage allowance and sward structure could be far from the possibility of farmers to follow at paddock level and depend on shade availability and water quality and availability. However, observing grazing times at key periods of the day could give clues to adequate grazing management.

Although foraging behavior studies have already been defined as important evaluations, the extent of the evaluations (e.g., during the daylight periods or 24 h periods) that best represent animal behavior remains uncertain. Both protocols were found in the international literature (daylight × 24 h). This is due to three main factors: (i) availability and costs of skilled observers; (ii) circadian behavior rhythms associated with daylight, especially in temperate climates; and (iii) the need for artificial light during the night.

The 24-h visual evaluations may currently be less feasible, in part, because they require a large number of trained people. Furthermore, artificial light during dark periods may affect the natural behavior of animals (12), especially if the animals are not very tame or not used to being under artificial light. Thus, many research groups are searching for accurate automatic recording methods of behavior (13). Nevertheless, continuous 24 h assessments are the most accurate for evaluations, and methods need to be calibrated for automatic recording and evaluating a longer and fixed period, regardless of the climatic season (1,440 min/day). On the other hand, evaluations performed only during daylight periods, regardless of the time interval, have been justified based on the pattern of ruminants' diurnal foraging behavior, especially for the main meals during the day, and have lower labor requirements (14, 15).

The problem lies in the autumn and winter assessments. In these climatic seasons, restriction to daylight evaluations could be seriously biased because of the reduced day length (photoperiod) and, more importantly, because during this period the quantity and quality of the forage are substantially different from that during other periods of the year, especially in natural grasslands. Moreover, daylight observations do not consider natural animal behavior. Usually, animals tend to graze at night (16), mainly in tropical and subtropical conditions. Preference for grazing at night could occur because of more comfortable air temperatures during this period (12), although nighttime grazing activity has a shorter duration compared with that during daylight. However, these grazing events could represent as much as 35% of the total grazing time over 24 h in hot weather or during long nights (17–19). Furthermore, during these foraging events at night, (19) demonstrated that animals have a heavy bite mass (19). This part of the day needs to be considered in assessments that consider animal welfare and evaluate the efficiency of the management system.

Thus, considering the importance of the daylight period on behavior and the interaction of the daylight period with temperatures in subtropical environments, the objectives of this work were to evaluate for how long foraging should be monitored over a 24-h period to predict foraging behavior of beef heifers within a season and determine the patterns of foraging activity over 24 h.

## Materials and Methods

### Local, Climate, Experimental Area, Area Management, and Behavior Assessments

The experimental area is located in the southern part of Brazil, Rio Grande do Sul state, with the center of the experimental area at ~29°43′30″S, 53°45′33″W. This area belongs to the Federal University of Santa Maria (UFSM). The local climate is classified as subtropical humid, with a mean ambient annual temperature of 19.2°C and a mean annual rainfall of 1,770 mm at 95 m above sea level (20). During the trial, the mean maximum temperature was 22.7°C and the mean minimum temperature was 17.1°C, the mean precipitation was of 130.6 mm per month, November is the wettest month (294.9 mm), and October is the driest (53.6 mm).

The experimental area was 22.5 ha, which was divided into six rectangular paddocks of 3.5 ha each. Each of these six areas was then subdivided into seven smaller sub-paddocks and managed with a rotational grazing method. The criteria that defined the rest period of sub-paddocks was the thermal sum accumulated (degree Celsius per day; degree day, DD) for the duration of leaf elongation of two functional groups of grasses (21) (*as described below*) that compose the swards of Campos grasslands.

To define the rest intervals of the rotational grazing method (original trial treatments), mean phyllocron (time in DD for complete leaf elongation) of functional groups A/B and C/D (375 or 750 DD) was multiplied by the number of expanding leaves per tiller, generating the rest periods of each sub-paddock. The number of expanding leaves of grasses in the functional groups is intrinsic to the genetic traits of plants and defines the time of rest intervals (21). Following this logic, over 3 years, three paddocks were managed using a rest interval of the accumulated thermal sum of 375 DD, and the other three paddocks were managed using 750 DD of the accumulated thermal sum. Therefore, the occupation period was defined by dividing rest intervals (in thermal sum) of each treatment by the number of sub-paddocks, less one (sub-paddock under occupation), resulting in the time, in degree Celsius, of occupation of each sub-paddock. The accumulated thermal sum to manage the paddocks generated a varying number of days for occupying the sub-paddocks, according to ambient temperature and weather seasons.

The 375-DD rest interval was based on the accumulated temperature for elongation of 2.5 leaves per tiller of grasses of functional groups A and B [e.g., *Coelorhachis selloana* and *Paspalum notatum*; (22)]. The 750-DD rest interval was based on the accumulated temperature for elongation of 1.5 leaves per tiller of functional groups C and D [e.g., *Aristida laevis* and *Saccharum trinii*; (23)]. Those species had an important contribution to sward composition of the area and, consequently, on available herbage mass.

Over 3 years, when measuring the rest interval effects, a total of 15 experimental evaluations of beef heifer grazing behavior were conducted. All assessments were performed with visual observations during 24 consecutive hours. In each year, a variable number between 24 and 36 beef heifers (at least four heifers per paddock) was evaluated with variable body weights (177–215 kg) and age (12–24 months) (Table 1). Variations in weight and age were within the range for heifers rearing to breed at 2 years of age.

**Table 1:** Mean initial body weight, age, and number of beef heifers monitored during a study to quantify the daily foraging activities.

	**2010^**a**^**	**2011^**b**^**	**2012^**b**^**
Body weight	215	177	185
Age	18	12	12
Monitored animals	24	36	24
Breed type	Angus	Angus; Charolais × Nelore	Angus
Date of behavior evaluations^c^	June 11	January 20	January 16
	August 15	April 09	March 24
	September 30	June 04	May 26
	December 17	July 19	July 07
		September 03	September 12
		November 18	

*Body weight is expressed as kg BW. Age is expressed as months. “Monitored animals” is the number of animals assessed in each evaluation year. Breed type represents the breed of animals assessed each year. Date of behavior evaluations represents the dates when the assessments were performed*.

a *From September to December*.

b *In 2011 and 2012, heifers started with 12 months remaining in paddocks until they reached 24 months*.

c *June, July, and August represent winter; September represents spring; December and January represent summer and March; April and May represent autumn*.

During the grass growing seasons over the 3 years (springs, summers, and autumns), heifers were only supplemented with mineral salt and had access *ad libitum* to freshwater. During the first winter (2010), heifers were supplemented with mineral protein salt *ad libitum* (24). During the second winter (2011), heifers were supplemented with grounded corn at a proportion of 0.5% of body weight (BW). During the third winter (2012), the heifers were supplemented with 0.5% of BW with wheat bran (85%) and glycerol (15%). In all winter seasons, except for the first when mineral protein salt had its intake limited by NaCl concentration, the intake was not higher than 200 g per animal. In the second and third winter, supplement did not exceed 0.5% of BW and was available only from 09:30 to 10:30 h, when grazing activity is lower. The stocking rate adjustments were made each 28 days using 4.5% of the herbage allowance (4.5 kg MS per 100 kg of BW) considering 70% of grass leaf blades creating part of the sward mass.

The experimental area was arranged in a randomized block design with the two rest periods as the treatments (375 and 750 DD), with three area replicates (six paddocks with sub-paddocks, three for each rest period), using rotational grazing management. The blocking criterion was the relief. Details regarding management can be found in Barbieri et al. (10).

Before this 3-year experimental period and 15 behavior assessments, behavior variables were tested through an analysis of variance model (using *P* ≤ 0.05 as the significance level). Because there were no differences between grazing and rumination time between 375 and 750 DD treatments, all data were used to form a larger database. Then, these databases were combined by year, generating four evaluations in the first year (4 × 6 paddocks = 24 replicates), six evaluations over the second year (6 × 6 = 36 replicates), and five evaluations in the third year (5 × 6 = 30 replicates). To this new analysis, the year was used as a block in the statistical model to remove possible climatic differences among the years. Next, data were clustered by season, regardless of year (blocked), which generated 12 replicates in summer, 24 replicates in autumn, 36 replicates in winter, and 18 replicates in spring. Each replicate evaluated at least four heifers. Even after clustering the data (years and climatic seasons), grazing and rumination time did not present significant differences between the 375- and 750-DD treatments, making it possible to pool all data to perform the timing and sample sufficiency analysis. The original data and animal performance can be found in Soares (25).

### Natural Grassland Characterization

The pasture that composes the Campos grasslands (6) presents a well-defined double layer canopy structure. In this case, the lower strata were composed of short-grass species, such as *Axonopus affinis* and *Paspalum notatum*, mostly with a prostrate growth pattern and were the major grasses in the above-cited A/B functional groups. These species were highly preferred by free-ranging cattle. In the upper strata were grass species with a tussock-like growth habit, such as *Andropogon lateralis* and *Aristida laevis*, the major grasses in the above-cited C/D functional groups (26). Moreover, the experimental area was mostly composed of C_4_ metabolic cycle grasses (above 75% of herbage mass). During the cool seasons, sward production was dramatically reduced concomitant with the decrease in its nutritional value.

The herbaceous vegetation of the area consisted (mean contribution for green herbage mass) primarily of *Andropogon lateralis* (±37%), *Aristida laevis* (±14%), *Saccharum trinii* (±6%), *Shorgastrum nutans* (±6%), *Paspalum plicatulum* (±3%), *Axonopus affinis* (±6%), *Paspalum notatum* (±9%); species within the Umbelliferae family, including *Eringium horridum* (±3%); and ±16% representing other plant families, including woody plants (each with negligible amounts; <1%). Furthermore, 117 species, representing 33 grass genera, have been documented in this experimental area (27). Species classified as A and B functional groups (*Andropogon lateralis, Axonopus affinis, and Paspalum notatum*) comprised 52% of the mean green herbage mass and those as C and D groups (*Aristida laevis, Saccharum trinii, Shorgastrum nutans, and Paspalum plicatulum*) comprised 29% of the herbage mass. These species and group contributions varied throughout the year, mostly because of the variations in environmental temperatures over time (seasons). See Cruz et al. (21) for details on functional groups. The quantity of senescent plant material also changed across seasons, being lower in the spring (±20% of total herbage mass) and greater in the winter season (±55% of total herbage mass). All these values (species contribution and botanical composition) were sampled using the BOTANAL method, as described by Tothill et al. (28).

Over the 3 years, herbage mass (HM) was measured, each 28–32 days, using a visual standard comparison, calibrated with a double sampling technique (29), with 20 visual samplings and six samples cut at ground level, using 0.25 m^2^ quadrats. All regression equations derived from visual assessments were above 0.7 determination coefficient (*R*^2^). In each evaluation of HM, sward height was measured with a sward stick at the same points as HM evaluations. We did not consider the tall tussock grasses in sward height measurements.

### Grazing Behavior

All 15 grazing behavior evaluations began on the second day of occupation of the sub-paddocks, regardless of whether the management was 375 or 750 DD (dates in Table 1). The mean time of occupation of the sub-paddocks was 4 days (range from 2 to 5 days in spring and summer) and 7 days (range from 5 to 10 days in autumn and winter). In all assessments, the experimental unit was the sub-paddock and the variables explored were the mean values of at least four tested animals.

Previously for each assessment, heifers were exposed to night observations with flashlights to acclimate them to this type of light and they were habituated to close handling by people using daily supplementation on grassland. Thus, flashlights and close observations appeared to have minimal effects on the behavior of the animals. In each evaluation, trained evaluators were placed at ground level in “easy-to-view” locations for heifer behavior recording. Four trained evaluators were used for each sub-paddock, taking turns every 2 h.

Total grazing, rumination, and other activity times were visually recorded, every 10 min over 24 consecutive hours, and the results were expressed in minutes per day. Considering that sub-paddocks were 0.5 ha, and animals expressed their behavior in groups, each observer was able to handle a single paddock observation. The recording frequency was chosen based on previous data reported by Gary et al. (30) and Mezzalira et al. (31). Grazing was defined as including time spent searching, selecting, and gathering (eating) forage, similar to that previously described by Hodgson (18). Rumination time was defined as the cessation of grazing and the beginning of jaw movements. Time of other activities was considered the time when animals were not foraging or ruminating and could be idle, engaging in social activities, drinking water, or eating supplements (32).

### Treatments for Timing, Sampling Sufficiency Evaluation, and Natural Behavior

As previously described, an analysis of variance showed no differences among treatments (375 and 750 DD rest intervals). Thus, data were recombined in five treatments regardless of the rest intervals. New treatments consisted of the comparison between timing and duration of observation periods to test the sufficiency of sampling duration for grazing time analysis and to determine the behavior of heifers in the natural environment. Again, each paddock area was used as a replicate in each season, generating 12 replicates in summer, 24 in autumn, 36 in winter, and 18 in spring. Differences among seasons were not compared because of the differences in day length among seasons and differences in green biomass availability and herbage quality.

**Figure 1:**
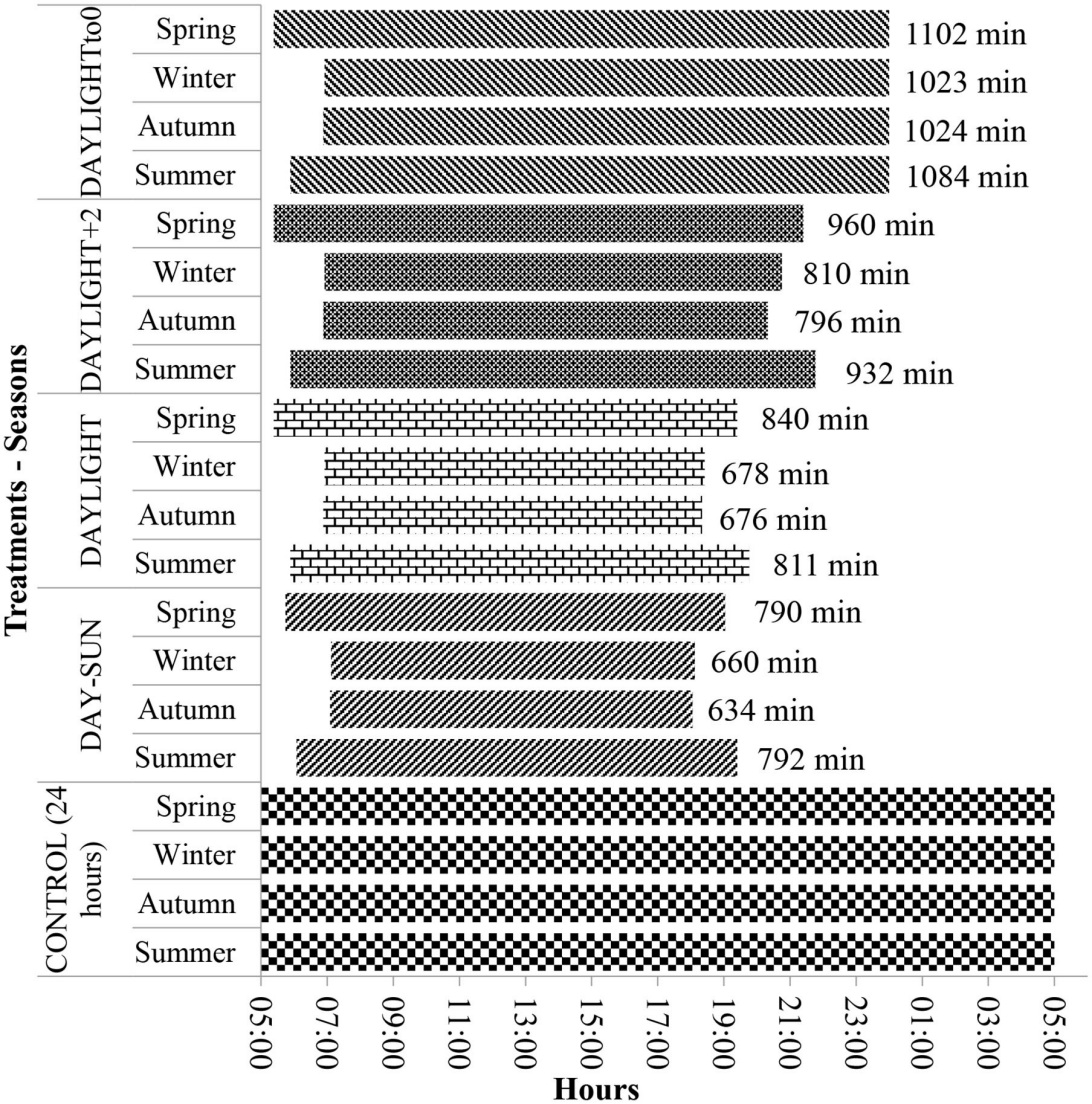
Graphical timeline representation of the timing and duration of evaluations of grazing behavior in 24 h (control; 1,440 min assessment) and the tested periods of time (treatments): DAY-SUN (sunrise to sunset), DAYLIGHT (down to nightfall), DAYLIGHT+2 (down to nightfall plus 2 h after dark), and MIDNIGHT to 0 (down until midnight).

Validation of the timing and duration of observation periods in each season accounted for grazing, rumination, and other activity times observed over uninterrupted periods of 24 h. Treatments consisted of the evaluation of four distinct periods having different lengths based on the following selected intervals: *sun duration*—during the day from sunrise to sunset (DAY-SUN); *daylight duration*—from dawn to dusk (DAYLIGHT); *DAYLIGHT plus 2 h* (DAYLIGHT+2); *DAYLIGHT to midnight* (DAYLIGHT to 0); and the *entire 24 h* (CONTROL) (details in Figure 1). All data were compiled by fractioning the CONTROL dataset.

To obtain the times of sunrise and sunset, historic data were used (mean of 30 years) registered by the National Institute of Meteorology (INMET) station, located 3 km from the experimental area. The mean time of sunrise and sunset was calculated for each season. Using these times, the beginning and the end of the DAY-SUN treatment were identified (Table 2). This information also was used to obtain the *dawn* and *dusk* durations [sun position 6° above (sunrise) and −6° below (sunset) the horizon (33)], and this time was added to the DAY-SUN treatment. The mean values of dawn and dusk (in minutes, mean of each season) were added to the mean sunrise and sunset hours for each season to determine the start and the end of behavior evaluations that defined the DAYLIGHT treatment (see Table 2).

**Table 2:** Mean hour of dawn, sunrise, sunset, and dusk among the four climatic seasons during a study to quantify the daily foraging activities of beef heifers.

**Event**	**Climatic seasons**			
	**Summer**	**Autumn**	**Winter**	**Spring**
Dawn	05:53	06:53	06:55	05:23
Sunrise	06:04	07:05	07:07	05:44
Sunset	19:24	18:03	18:07	19:02
Dusk	19:46	18:20	18:25	19:24

*See the text for the definitions of dawn, sunrise, sunset, and dusk. Mean hours of the events are an average of the last 30 years, provided by the National Institute of Meteorology (INMET)*.

In addition to these observation periods, to determine if animal behavior was being represented, other evaluation periods were observed. In the DAYLIGHT+2 treatment, animal evaluations were considered from dawn until 2 h after dusk ended. For the DAYLIGHT to 0 treatment, behavior was compiled between dawn and midnight (00:00; midnight). For DAYLIGHT+2 and DAYLIGHT to 0 treatments, the end of the evaluations was considered a fixed period of time. The evaluation period (time), even within the same treatments (except in the CONTROL treatment), was different between climatic seasons, and this occurred because of photoperiod changes among climatic seasons (Figure 1), influencing the time the assessments began.

### Behavior Data Analysis

The statistical analyses used was a block design model where each year was considered the block. Each paddock was considered a replicate (mean of animals inside the paddocks) and there were six replicates (paddock number in the experimental area) in each trial (15 trials for 3 years). In spring, data were analyzed with 18 replicates, in summer with 12, in autumn with 24, and in winter with 36 replicates. Results are presented separately by season because of the differences in day length among seasons.

The analysis of grazing time (minute per hour during 24 h) was performed using the mean values of grazing time from all replicates in the database. For this analysis, data were separated by climatic seasons, and using mean values of all replicates in each climatic season, the grazing time (minute per hour) was calculated for each hour of the day. From this, the grazing time in each hour between climatic seasons was compared.

Initially, data were submitted to a Bartlett test followed by a Shapiro–Wilk test to determine the homogeneity of variances and normality of residuals, respectively. After confirming this, the data were submitted to an analysis of variance and *F*-test. Mean comparison analyses were conducted using PROC MIXED (Tukey test) in SAS 9.2 software, including the model effects of blocks (years) and treatments (evaluation periods). The criteria for sampling sufficiency of the duration of observation periods were defined as occurring when comparisons between the CONTROL and treatments were similar. For all statistical tests, significance was defined as *P* < 0.05.

## Results

The main objective of this article was to evaluate the extent of observations of grazing behavior over a 24-h period to accurately represent this behavior, so we will discuss our data considering at the same time feasibility and representativeness to predict foraging behavior mediated by pasture management practices.

### Sward Characteristics

Mean herbage mass maintained during the experimental years was 3,871 kg DM/ha, ranging from 3,017 to 4,242 kg DM/ha. Furthermore, mean sward height, without tussock species, was 20 ± 3.9 cm, ranging from 17.3 ± 3.3 to 22.5 ± 4.1 cm. Sward characteristics were similar among the paddocks and typical of this grassland formation.

### Timing and Duration of Observation Periods

There were differences (*P* < 0.05) for grazing, rumination, and other activity times among all seasons and treatments within 24 h (Table 3). There were differences in grazing time even among lower observation period treatments (DAY-SUN vs. DAYLIGHT) in summer and winter seasons. Furthermore, these treatments presented lower (*P* < 0.05) foraging times than did the time observed in the CONTROL. In summer, grazing time measured in the DAY-SUN treatment represented only 82.7% of the total time spent grazing over 24 h and this treatment evaluated 56.9% of day length. In DAYLIGHT, grazing time observed represented 88.1% of the total grazing time and the DAYLIGHT evaluated 60.4% of day length.

**Table 3:** Grazing, rumination, and other activity times of beef heifers in a natural grassland managed under rotational grazing among the four climatic seasons.

	**Evaluation periods (treatments)**					STD*	***P*-value**
**Min/day**	**CONTROL**	**SUN-DAY**	**DAYLIGHT**	**DAYLIGHT+2**	**DAYLIGHT to 0**		
**Summer**							
Grazing	648^a^	536^c^	571^b^	597^b^	634^a^	11.2	0.001
Rumination	517^a^	191^d^	196^d^	267^c^	321^b^	13.8	0.001
Other act.	275^a^	93^c^	103^c^	126^bc^	155^b^	13.8	0.001
**Spring**							
Grazing	692^a^	549^c^	575^c^	609^b^	633^a^	14.7	0.001
Rumination	473^a^	164^d^	176^d^	230^c^	311^b^	10.2	0.001
Other act.	275^a^	97^c^	109^c^	141^b, c^	156^b^	12.4	0.001
**Autumn**							
Grazing	637^a^	449^c^	475^c^	521^b^	602^a^	12.9	0.001
Rumination	469^a^	114^d^	122^d^	176^c^	270^b^	9.2	0.001
Other act.	334^a^	107^c^	113^c^	133^c^	178^b^	10.4	0.001
**Winter**							
Grazing	597^a^	447^d^	476^c^	502^b^	566^a^	7.7	0.001
Rumination	437^a^	91^d^	95^d^	167^c^	303^b^	8.1	0.001
Other act.	406^a^	142^d^	149^c, d^	171^c^	191^b^	9.1	0.001

*See text for definition of the different treatments (Control, SUN-DAY, DAYLIGHT, DAYLIGHT+2, and DAYLIGHT to 0). Grazing, rumination, and other activities (Other act.) are expressed in minutes. Different lowercase letters in a line differ by Tukey test at 5%*.

* *standard mean deviation*.

In winter, the DAY-SUN treatment represented only 47.2% of day length and covered 74.9% of the grazing time observed in the CONTROL. In the DAYLIGHT treatment, 79.7% of the foraging time of the CONTROL was represented and 50% of the day length was observed. Rumination and other activity times were similar between DAY-SUN and DAYLIGHT treatments among all seasons. However, rumination time was lower in these treatments relative to that of the CONTROL.

In the DAYLIGHT+2 treatment, grazing time differences were observed in the spring, autumn, and winter when compared with other evaluation periods. In this treatment, grazing time was greater than the time spent grazing in the two treatments that evaluated grazing time only during the day clarity period (DAY-SUN and DAYLIGHT) and lower than the grazing time observed in DAYLIGHT to 0 and CONTROL treatments. In spring, DAYLIGHT+2 evaluated 68.1% of the day length and grazing time represented 88% of the CONTROL. During autumn, this treatment evaluated 57.5% of the day length and 81.5% of the grazing time in the CONTROL. During winter, DAYLIGHT+2 represented 84.1% of the CONTROL grazing time, evaluating 58.4% of the day length. Regardless of the season, the DAYLIGHT+2 treatment reached the grazing time representativeness of time grazing by the heifers observed over 24 h. In general, rumination and other activity times increased with the increase in the evaluated period during all seasons.

Grazing time only began to be represented when a large portion of the night was added to the evaluation period. Sampling sufficiency of the duration of the observation period was achieved when the evaluations were undertaken until midnight (DAYLIGHT to 0 treatment) in the four seasons (treatment DAYLIGHT to 0 vs. CONTROL; summer *P* = 0.485; spring *P* = 0.278; autumn *P* = 0.212; winter *P* = 0.196).

Using this evaluation period, during summer, 77.1% of the day length with 97.8% of the CONTROL grazing time was used. In spring, the evaluation period had 76.4% of day length and achieved 91.5% of the foraging time observed during the CONTROL period. During winter, grazing time in the DAYLIGHT to 0 represented 91.5% of grazing activity of the CONTROL, evaluating 72.9% of the day length. In autumn, grazing time in the DAYLIGHT to 0 represented 94.8% of the activity of the CONTROL, evaluating 73.6% of the day length.

Considering the time spent in rumination and other activities, even with the increase in the observation periods, differences were observed when compared with that of the CONTROL (*P* < 0.05). Overall, in summer and spring, rumination time during periods of natural clarity (day) represented only 37.6% of rumination time compared with the 24-h period (CONTROL). The remaining rumination time (62.4%) was observed during dark periods (night). In the cool seasons (autumn and winter), 23.9% of rumination time was observed during light periods and 76.1% overnight. Furthermore, the remaining activities followed the same pattern; during summer and spring, 31.1% of other activities occurred during the day (natural light), whereas the remaining time (68.9%) was observed during darkness. In the cool seasons (mean of autumn and winter), 43% of other activities were distributed in the day and 56.9% during darkness.

### Diurnal Foraging Patterns

**Figure 2:**
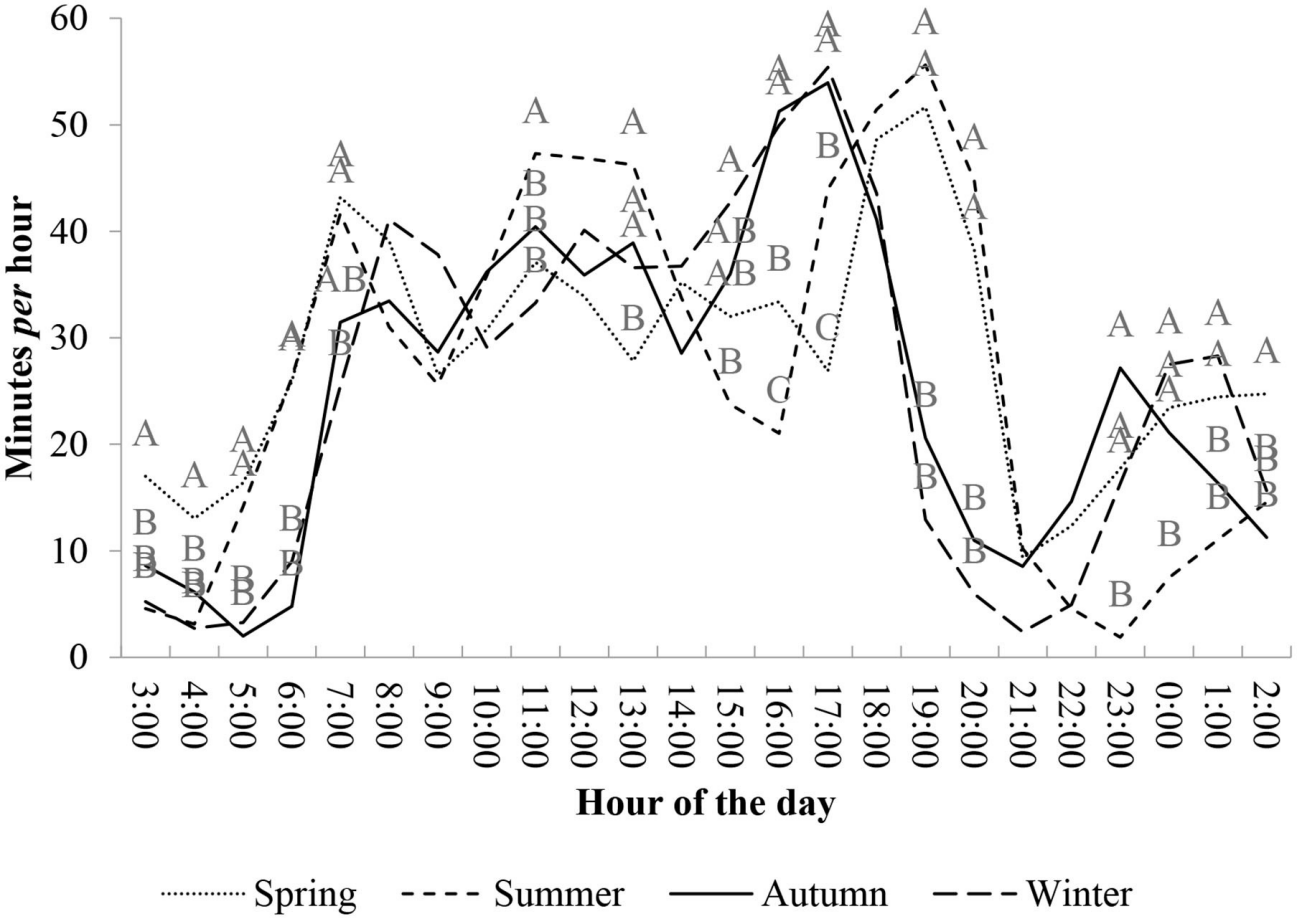
Mean foraging time (minutes per hour) of beef heifers, over 24 h, managed in natural grassland under rotational grazing method among the four climatic seasons over the years of 2010–2012 (*different capital letters in a column differ by Tukey test at 5%).

Grazing time distribution over 24 h presented some similarities among seasons, mainly when comparing among warm seasons (summer and spring) and cooler seasons (autumn and winter) (Figure 2).

During warm seasons, the first intense grazing cycle (or peak) occurred earlier than in cooler seasons, at ~04:00 in the morning. At 05:00, grazing activity was more intense during warm seasons than during cool seasons (*P* < 0.05). In cooler seasons, the first grazing peak started at ~06:00 h. The difference (*P* < 0.05) in grazing intensity (time spent grazing per hour) between warm and cool seasons was observed until 08:00. In all seasons, after this intense activity, grazing activity was reduced until 10:00 h (more details in the Supplementary File; Table 4).

**Table 4:** Hourly mean foraging distribution of beef heifers over 24 h foraging behavior assessments in a natural grassland managed under rotational grazing.

**Timetable**		**Time foraging (min/h)**				**Standard error**
		**Autumn**	**Winter**	**Spring**	**Summer**	
00:00	01:00	21.1^a^	27.5^a^	23.4^a^	1.8^b^	3.45
01:00	02:00	16.4^b^	28.3^a^	2.4^a^	7.5^c^	2.97
02:00	03:00	11.3^b^	16.7^b^	24.7^a^	11.0^b^	2.76
03:00	04:00	8.6^b^	5.2^b^	16.9^a^	14.6^a^	2.7
04:00	05:00	6.1^b^	2.7^b^	13.1^a^	4.6^b^	2.3
05:00	06:00	2.0^b^	3.3^b^	16.4^a^	3.1^b^	3.21
06:00	07:00	4.8^b^	9.0^b^	25.9^a^	14.2^a, b^	4.13
07:00	08:00	31.5^b^	25.4^b^	43.1^a^	26.3^b^	4.4
08:00	09:00	33.5	41.1	39.1	41.6	3.47
09:00	10:00	28.6	37.8	26.6	31.1	3.64
10:00	11:00	36.2	29.1	30.7	25.6	4.11
11:00	12:00	40.4	33.3	37.1	35.8	3.38
12:00	13:00	35.9	40.1	33.9	47.4	3.61
13:00	14:00	38.9^a^	36.6^a^	27.8^b^	46.8^a^	2.48
14:00	15:00	28.5^b^	36.7^a, b^	35.2^a, b^	46.3^a^	2.41
15:00	16:00	36.0	42.7	31.9	33.7	2.84
16:00	17:00	51.2^a^	49.9^a^	33.4^b^	23.7^b^	3.33
17:00	18:00	53.9^a^	55.4^a^	26.9^b^	21.1^b^	4.47
18:00	19:00	41.1	43.6	48.6	43.9	3.02
19:00	20:00	20.6^b^	12.9^b^	51.7^a^	51.4^a^	4.95
20:00	21:00	10.9^c^	5.9^c^	38.3^b^	55.6^a, b^	5.43
21:00	22:00	8.5^b^	2.4^c^	9.4^b^	44.8^a^	4.19
22:00	23:00	14.6	4.9	12.3	10.2	2.43
23:00	00:00	27.2^a^	16.3^a^	17.3^a^	4.6^b^	3.84

*Time foraging is expressed in minutes for each daily hour. Different lowercase letters in a line differ by Tukey test at 5%*.

During late morning, at ~11:00, a second intense peak of grazing activity occurred in the summer and it was different from that of other seasons (*P* < 0.05). In this same time of the day (late morning and early afternoon), cool seasons and spring had low intensity and more constant grazing distribution. Regardless of the season, during late afternoon, and at the beginning of the night (16:00–20:00 h), a second intense grazing peak was observed. This peak in grazing activity started earlier in the cool season as compared with that of warm seasons. In winter and autumn, this intense grazing activity started at ~15:00–16:00 h. This grazing peak had, approximately, a duration of 3, 1 h less than the duration of the grazing peak observed during the warm season. In summer and spring, the intense foraging activity occurred between 17:00 and 21:00 h.

After this grazing peak in the late afternoon, grazing activity was reduced during the early evening. In cool seasons, this grazing activity reduction ranged from 19:00 to 22:00 h. During spring, this reduction was shorter and ranged from 21:00 to 22:00 h, and in the summer, grazing activity was evident from 21:00 to 0:00 h. Furthermore, in spring, autumn, and winter, heifers had another short grazing peak during the night (between 23:00 and 01:00 h). Only during summer did heifers present low foraging activity during the night.

## Discussion

During the 3 years when grazing behavior was evaluated, herbage mass and sward height did not present values in the range of sward structure considered limiting for beef heifer intake on natural grasslands (1). Thus, the similarities allowed us to assume that the grazing behavior of beef heifers in this study was not influenced by these factors.

Recently, animal behavior evaluations (or trials) have focused on the observation periods during daylight observations, regardless of the climatic season or pasture type (8, 11, 34, 35). This observation period was based on the major grazing events featured by the animals by their natural behavior, mostly observed in temperate climate conditions. In such situations, the weather is characterized by milder environmental temperatures during the daylight period and cold environmental temperatures during the night (14). Thus, because of thermal comfort, grazing activity occurs predominantly during daylight hours (15) and major grazing events occur near sunrise and sunset, with the latter having greater intensity and longer duration (36).

However, in tropical and subtropical conditions, the temperature distribution is different over a 24-h period, especially in different seasons, and as seen in our results, to maintain animal welfare, animals distribute their grazing activities differently, having more dispersed foraging activities over 24 h as compared with those in temperate climates. In support of this, in subtropical and tropical climates, animals can conduct a significant portion of grazing during non-daylight hours together with rumination and resting (12, 19, 37).

Another important feature related to diurnal ingestive preferences of animals in temperate climates is the high nutritional quality of C_3_ grass species, a typical trait of the pastures in temperate climates that easily supports animal nutritional demands over the day when the sward is high or herbage mass allows high bite masses. Because of this, night foraging activity is usually characterized as occurring in shorter intervals and less intense bouts. Overall, night foraging represents a small percentage of daily foraging time and contribute minimally to daily herbage intake in temperate climates (16, 38). However, in subtropical natural grasslands, as in our experiment and as observed by Trindade et al. (11), sometimes the nutrient concentrations of the pasture are poor, and consequently, animals have to spend more time during the day to attend to their energetic requirements, even with no limiting intake factors based on sward conditions (high or mass).

Thus, evaluations of grazing time, which consider only day length (DAY-SUN and DAYLIGHT), are incomplete in the representation of grazing time and natural behavior of heifers over 24 h (CONTROL). In these situations, grazing time was significantly lower than grazing time normally presented by the animals. Even when 2 h was added past sunset (DAYLIGHT+2), the time spent grazing was significantly lower than grazing time measured over 24 h. This definitively suggests that there is significant nighttime foraging (Figure 2). Champion et al. (14) and Gregorini et al. (12, 19) suggest that both sheep and cows may have significant meals at night. In temperate climates, ruminants have approximately three major grazing events per day: at sunrise, around 12:00, and sunset (36). However, this pattern is flexible and affected by external environmental conditions, especially environmental temperatures. According to Gregorini et al. (12), an adaptation could be an increase in the length of grazing events and a decrease in the number of meals during short days, or ruminants could increase meal numbers, including times at night to allocate these meals.

To faithfully represent the natural behavior of heifers, it is possible to confirm that the time extent of grazing behavior assessments that should be evaluated needs to include part of the night. In our case, despite being 35 min less total grazing time than the CONTROL, evaluations using all day and until midnight showed no differences (*P* > 0.05) when compared with the natural behavior of heifers. To achieve this representativeness, we evaluated 75% of the entire day.

Additionally, our data support that in a tropical climate situation, sunlight (including dawn and dusk) has a strong influence on animal activity (39), even in warm environments. Furthermore, another important fact is that grazing events, which occur after sunset, should not be underestimated (38). Nevertheless, trials assuming that grazing time observed only between sunrise and sunset (natural light) represents an accurate estimate of the grazing time are underestimating the real time that animals spend foraging. This bias can be magnified when this incomplete information is used to estimate/calculate other behavior variables (e.g., bite mass) causing serious misunderstandings, especially in trials were the bite mass is estimated by the division of daily animal intake by the daily bite number, which, in turn, is estimated by multiplying the bite rate by total grazing time.

Of course, to determine the “size” of the evaluation period that must be performed, baseline experiments need to be conducted. In our case, it was possible to reduce the total evaluation period by 25% with no effects in grazing representativeness [all seasons mean: 94% of the total grazing time observed in the CONTROL treatment (*P* > 0.05)]. This protocol reduces possible overestimations of other dependent variables of grazing time and allows the comparison among trials conducted in similar conditions, mostly by representing the actual grazing time of animals.

Grazing activity occurs mainly during daylight and the influence of day length changes the foraging patterns of animals (Figure 2). Moreover, the different grazing peaks during different seasons demonstrate the ability of animals to adapt their ingestive activity to variations in daylight, reserving most rumination and rest activities for periods of darkness to maintain their welfare. Furthermore, there are other factors to determine this pattern, such as the difficulty of food selection during dark periods (15), defense mechanisms (40), and hormonal factors (41).

The extent of grazing taking place during daylight in summer and spring (higher temperatures) compared with that in autumn and winter (lower temperatures) was not highly variable, even though the peaks in this behavior occurred during different periods of the day. In summer and spring, grazing begins earlier in the day compared with that of autumn and winter seasons. Consequently, grazing peaks during the morning are more intense in warm seasons than cool ones. This probably occurs because of the longer photoperiod, which encourages the animals to begin foraging earlier (12, 19), and thus, it reduces the need for foraging during the hottest period of the day (late morning/early afternoon). After the first meal (morning grazing peak), animals decrease the time spent grazing, probably because of rumen filling (3).

Another important practical information indicated by our data is related to the use of feeding supplements in production systems. When the use of supplementary sources of feeding is necessary, supplements should be offered to the animals between the grazing peaks. In our subtropical environment, this means offering it from 08:30 to 09:30 (spring–summer) and from 09:30 to 10:30 (autumn–winter) (Figure 2). Thus, using this information, it is possible to reduce herbage substitution by the supplement, and this was already used in that particular environment (42, 43). Furthermore, when energetic supplements are used, this management schedule allows better use of herbage nitrogen (44).

Grazing peaks earlier in the afternoon during autumn and winter compared with that of summer and spring, which may be a consequence of the interaction of photoperiod and environmental temperatures. The first is related to the light period when animals can distribute themselves for better grazing activity (15), avoiding the high-temperature periods of the day. Secondly, animals start grazing when temperatures are milder (late afternoon). In seasons with high environmental temperatures, this grazing peak [mainly in summer (12, 19)] is slightly longer than in other seasons.

The longer duration and later start of the afternoon grazing peak probably influences the later onset of grazing during the nighttime period during summer. Only during summer did animals not obtain a meal during the night between 22:00 and 01:00 h. Grazing events over the night are also necessary for the animals to maintain their metabolic heat production (by rumen fermentation) during cool seasons (45). Furthermore, our data of nighttime behavior observations contradicted the assumption that heifers do not forage for significant periods at night (16, 37, 38). Therefore, if one of the experimental goals is to measure the length of foraging events and represent natural grazing activity, it is necessary to accurately evaluate periods of nighttime grazing, especially under subtropical and tropical climate conditions.

## Conclusion

Visual observations beginning at dawn until midnight represented the total foraging time in a subtropical natural grassland. This period could be used to represent grazing activities performed during 24 h, as well to evaluate the natural behavior of heifers, and this would be useful for the calibration of automatic recording devices.

Diurnal evaluations of grazing behavior of beef heifers do not represent the necessary time to represent grazing activities in natural grasslands in subtropical and tropical conditions.

Beef heifers managed in natural grasslands have a diurnal pattern of grazing. However, there are significant grazing events in dark periods, and there are also significant changes between seasons in the times that animals perform these events. Farmers could use this daytime pattern to establish key periods of the day for observations of grazing behavior, such as the end of the morning grazing peak or the beginning of the evening peak.

## Data Availability Statement

The datasets generated for this study are available on request to the corresponding author.

## Ethics Statement

The animal study was reviewed and approved by Animal ethics committee—Federal University of Santa Maria.

## Author Contributions

FJ, ES, and LO compiled and organized the data, ran the statistical analyses, wrote the article, and assisted in the behavioral evaluations. BK, PC, and LM were responsible for the trials wherein data were collected, as well work conducted on the behavior assessments. FQ revised the manuscript. All authors contributed to the article and approved the submitted version.

## Conflict of Interest

The authors declare that the research was conducted in the absence of any commercial or financial relationships that could be construed as a potential conflict of interest.
